# Repercussion of COVID-19 Pandemic on Brazilians’ Quality of Life: A Nationwide Cross-Sectional Study

**DOI:** 10.3390/ijerph17228554

**Published:** 2020-11-18

**Authors:** Isabella Teotônio, Mariana Hecht, Luiz Claudio Castro, Lenora Gandolfi, Riccardo Pratesi, Eduardo Y. Nakano, Renata Puppin Zandonadi, Claudia B. Pratesi

**Affiliations:** 1 Interdisciplinary Laboratory of Biosciences and Celiac Disease Research Center, School of Medicine, University of Brasilia, Brasilia 70910-900, Brazil; isabella.msn.rocha@gmail.com (I.T.); marianahecht@gmail.com (M.H.); Lcgcastro@unb.br (L.C.C.); lenoragandolfi1@gmail.com (L.G.); pratesiunb@gmail.com (R.P.); 2 School of Health Sciences, University of Brasilia, Brasilia 70910-900, Brazil; 3 Department of Statistics, University of Brasilia, Brasilia 70910-900, Brazil; eynakano@gmail.com; 4 Department of Nutrition, School of Health Sciences, University of Brasilia, Brasilia 70910-900, Brazil; renatapz@unb.br; 5 College of Population Health, University of New Mexico, Albuquerque, NM 87131-0001, USA

**Keywords:** Brazilian population, COVID-19, pandemic, quality of life

## Abstract

The COVID-19 outbreak, caused by Sars-Cov-2, was officially declared a global pandemic in February 2020, after an unexpected increase in hospitalization and mortality. When faced with this new disease, social and physical distancing and quarantine emerged as solutions to reduce virus transmission. This article examines the quality of life (QoL) of the Brazilian population’s during this period of isolation, due to the COVID-19 pandemic by analyzing; physical, psychological, social, and economic aspects. An online survey was distributed from 27 May to 14 August of 2020. A total of 1859 surveys were completed. Our results indicate that Brazilians were more affected by economic and social aspects than psychological and physical. Unemployed participants and individuals who tested positive for COVID-19 presented the lowest QoL. Females showed worst QoL scores than males, but having children did not influence the score. Higher educational level was associated with a better perception of QoL. Not following social distancing guidelines presented better scores in the psychological domain than the ones following restrict or partial social distancing rules. This study is the first to evaluate adults’ QoL related to the Sars-Cov-2 pandemic in Brazil at a national level. Our data may help health authorities identify the main factors affecting the QoL of the Brazilian population, thereby orientating them to recover after the pandemic.

## 1. Introduction

The world population has been greatly affected by the Sars-Cov-2 (Coronavirus Disease 2019—COVID-19) pandemic, and the related economic, social, psychological, environmental, and public health consequences. The disease brought the risk of death from infection along with unbearable psychological pressure to people in the world [1]. By 14 August of 2020, there were 20,730,456 cases of COVID-19, and 731,154 deaths were registered in the world [2]. Almost half of the official cases (*n* = 11,109,443 cases; 53.6%) and deaths (*n* = 402,734 deaths; 53.61%) occurred in Americas. In South America, Brazil is the country with the largest number of registered cases of COVID-19 (63.7%, *n* = 3,164,785, until 14 August, 2020) and deaths (62.9%, *n* = 104,201 in the same period) [2,3]. Unfortunately, these numbers could be higher, due to the lack of testing and diagnosis capability at the beginning of the pandemic.

The relationship between the perception of vulnerability (i.e., unemployment, food, and job insecurity) and low quality of life (QoL) is widely accepted, however there are relatively few studies that link the current pandemic to reduced QoL in the general population of Brazil [4,5]. Taking into account the relevance of QoL, it is necessary to consider the consequences of the pandemic for the population [6,7]. Therefore, it is imperative to understand how the population impacted, especially in countries severely affected by the pandemic [8]. In Brazil, people are experiencing known risk factors for depression and anxiety, such as social isolation, food insecurity, unemployment, and or family income reduction. The economic uncertainty both during and after the pandemic aggravates these factors [9]. Consequently, this scenario of fear and uncertainties can affect the individuals’ perceptions of QoL.

Studies on this pandemic’s psychological impact on patients, medical staff, children, and older adults have been executed [1,10,11,12]; however, no study has looked into the QoL in the Brazilian population during these difficult times [7]. This study aimed to evaluate the Brazilian population’s QoL during the pandemic and the subsequent restrictive measures established to curb the infection rate. We expect to identify the main factors influencing the Brazilian population’s QoL during the pandemic to help in the recovery. Although there is no data on the QoL of the Brazilian population before the pandemic for comparison, knowing the population’s QoL during the pandemic will allow for future research to compare the current pandemic and possible future pandemics. Results can also make possible future comparisons with post-pandemic data showing how the pandemic affected the population.

## 2. Methods

### 2.1. Study Design and Instrument

This nationwide cross-sectional study was performed using a self-administered instrument (Appendix A) to investigate individuals’ QoL during the Sars-CoV-2 pandemic in Brazil. Our instrument was based on the World Health Organization Quality of Life Instruments (WHOQOL-BREF) Brazilian-Portuguese version [13], and the phrase “during the pandemic” was added to the questions to fit the current situation. Moreover, due to the pandemic’s economic impact, five questions to assess the respondents’ economic well-being composed the instrument (Appendix A), and the participants were also asked if they were diagnosed with COVID-19 (positive PCR-test). Therefore, the instrument was composed of four domains (with five questions each) to evaluate the QoL (physical, psychological, social, and economic). Each item was evaluated using a 5-point Likert scale (1 being the worst perception of QoL and 5 the best). Therefore, each domain could range from 5 to 25 points. Socioeconomic and demographic characteristics, such as gender, age, marital status, the state where responders currently reside were also included to compare the QoL among these variables. 

The instrument was placed on the online platform SurveyMonkey^®^ from 27 May to 14 August of 2020. Volunteers received the questionnaire’s link, inviting them to participate through nationwide recruitment done through email, messaging apps, and social networks. 

### 2.2. Participants and Ethics Approval

This study was performed with a convenience sample, where individuals from the entire country were recruited to participate. Researchers wanted to evaluate the QoL during the Sars-CoV-2 of the Brazilian population. Ethical approval was obtained for this study by the Ethics Committee University of Brasília (CAEE 30555220.3.0000.0008). The study followed the guidelines established by the Declaration of Helsinki and followed the Conduct, Reporting, Editing, and Publication of Scholarly work in Medical Journals. Once the respondents received and opened the questionnaire’s link, the inclusion criteria were explained. The inclusion criteria for participation were; participants had to be Brazilian, living in Brazil, aged 18 years or older, and agree to participate in the study before opening the questionnaire. Those that did not agree to participate were directed to a page thanking them for their time; while participants that agreed were directed to the survey. 

### 2.3. Statistical Analysis

Analysis of the data was done utilizing the GoogleForms^®^ tool and IBM SPSS Statistics for Windows version 22 (IBM Corp, Armonk, NY, USA). The statistical analysis was carried out, where higher scores indicate a higher QoL. Questions left blank were substituted by a median value of the corresponding dimensions. The total score was calculated for each participants’ characteristics. If more than 20% of the questions were left blank, the questionnaire was eliminated from the analysis.

The descriptive statistics (mean, median, standard deviation, floor effect, and ceiling effect) of the questionnaire’s subscales were presented. The within-subject comparisons of domain means were performed by one-way repeated measures ANOVA followed by Bonferroni’s post-hoc tests. A Student’s *t*-test and Variance Analysis (ANOVA) followed by Tukey post-hoc analysis was used to compare the domains’ values with the socioeconomic and demographic variables. All tests considered two-tailed hypotheses with a significance level of 5%. 

### 2.4. Factor Validity

The factor validity was verified by confirmatory factor analysis. The Chi-squared test of minimum discrepancy (chi2), the Root Mean Square Error of Approximation (RMSEA), and the Comparative Fit Index (CFI) evaluated the factor validity [14]. Both RMSEA and CFI ranges from 0 to 1 (RMSEA = 0 and CFI = 1 indicate a perfect fit). The model fit is accepted when RMSEA is less than or equal to 0.05 [15], and CFI is greater than or equal to 0.9. The four domains presented a good fit in the confirmatory factor analysis (RMSEA = 0.0068 (95% CI: 0–0.0157); CFI = 0.9992 and chi2 = 109.63; df = 101; *p* = 0.262) [14,16].

## 3. Results

From 27 May to 14 August of 2020, 1877 respondents accessed the questionnaire, and of those, 1859 individuals from all five regions in Brazil (North, Northeast, Midwest, South, and Southeast) that met the inclusion criteria, answered the questionnaire. Most of participants were female (*n* = 1349; 72.56%); white (*n* = 1096; 60.88%); with children (*n* = 966; 53%); living in urban areas (*n* = 1771; 95%); have post-graduate degrees (*n* = 993; 53.4%) and a half of them (50.32%) age < 39 y/o., and 95% of them live in urban areas. The minority were unemployed (*n* = 130; 7%) (Table 1). During the collection, most participants (*n* = 1800; 97%) did not test positive for Sars-Cov-2. There was no significant difference between the percentage of COVID-19 prospects with demographic and socioeconomic variables, except for educational level, where postgraduates had a higher positive percentage (Supplementary file—Table S1). Many participants had a family member who tested positive (*n* = 300; 16%). A small number of participants did not follow social isolation measures (*n* = 1.8%) (Table 1).

In general, females presented worse QoL than males (*p* < 0.05), except for the economic domain, which was similar (Table 1). Individuals age ≥ 40 y/o presented better QoL for all domains. Individuals with partners presented better QoL scores (except for the social domain—which was similar) to those without a partner. White individuals presented better QoL scores (both totals and domains) than brown and black respondents. Having children did not influence the general QoL, but individuals with children presented a lower perception of QoL regarding the economic domain than the ones without children. In most cases, a higher educational level was associated with better QoL, considering the psychological and physical aspects.

Unemployed individuals presented lower QoL scores (both total and each domain). Individuals that tested positive for COVID-19 presented the lowest QoL scores (except for the economic domain). Likewise, the QoL was affected (except for the social domain) when a family member was diagnosed with COVID-19. Experiencing the disease or having a family member experience it can unearth feelings of fear of possible health consequences related to the disease and also fear of death, consequently impacting the QoL perception. Individuals not following social distancing rules presented better scores in the psychological domain than those that follow restrict social distancing rules or those that leave home only for essential purchases. However, those results were similar to those that leave home for work, family visits, and essential purchases. Regarding the social and physical domains and total score, the individuals not following social distancing rules only differed from the ones that followed social distancing rules, but leave for essential purchases and has a family member that leaves the house for work (Table 1).

The total score Cronbach alpha values for psychological, social, and economic domains scores were >0.6, demonstrating good internal consistency. The QoL during the pandemic was most affected by economic and social aspects (lower means evaluated by one-way repeated measures—ANOVA followed by Bonferroni’s post-hoc tests) followed by the psychological and physical, respectively (Table 2). 

## 4. Discussion

This study is the first to evaluate the Brazilian adult population’s QoL related to the Sars-Cov-2 pandemic. During the data collection period (27 May to 14 August of 2020), the numbers of cases and death by COVID-19 were increasing in Brazil. During this research period, Brazil had already registered more than 3 million confirmed cases, and more than 100 thousand deaths by COVID-19 [2]. However, the number of people with COVID-19 is possibly nine to fifteen times higher than reported, due to the lack of diagnostic tests [17,18,19]. 

Our data indicate that the QoL during the pandemic period was most affected by economic and social aspects, followed by the psychological and physical factors. The results related to the economic domain were expected since the pandemic in Brazil aggravated an already difficult economic period, consequently affecting the QoL. Uncertainties about the future, such as work conditions, income, and social protection, lowered the mean scores for the economic domain of QoL. Moreover, the pandemic period in Brazil was marked by family income reduction and unemployment [20]. Despite that, most of the participants (*n* = 1458, 79%) were employed during the survey. However, during the pandemic, many families quarantined at home, increasing household expenses, such as water, energy, food, medicines, and other bills, which can influence the QoL, despite reducing their expenditure on other non-essential categories, such as office supplies and beauty products [21,22,23]. A small number of participants did not follow social distancing rules (*n* = 33; 1.8%), potentially influencing the social domain of QoL. However, only 125 (6.7%) followed social distancing, not leaving home to work, visit family members, or essential purchases. The individuals not complying with social distancing guidelines presented better scores in the psychological domain than those that followed these guidelines, as well as those that followed the guidelines and only left their home for the absolutely essential. In addition to social isolation, the psychological domain can be affected by the fear of contracting COVID-19. It could be speculated that because the Brazilian president repeatedly stated that COVID-19 was like the flu, that a portion of the Brazilian population was not worried about the possibility of contracting COVID-19. 

Social isolation measures were implemented in an attempt to prevent widespread community transmission. Most Brazilian cities closed restaurants, bars, night clubs, gyms, parks, shopping malls, and large events and gatherings were suspended while data was collected. Therefore, even those not following social distancing rules were restricted in their activities and the number of people they interacted with [24]. Additionally, these measures had critical socioeconomic implications, which affected the QoL of many, since these measures severely interfered with the outflow of industrial products and commodities, reduced local purchases, which interfered with people’s daily lives [24]. 

Data suggests that female respondents presented a worse perception of QoL than male respondents. In Brazil, women play a central role in food purchase and preparation for the family and are frequently responsible for household food decisions and housework [25,26]. While social distancing rules were in effect, women were responsible for the bulk of domestic chores, child-rearing, and education, in addition to their jobs, impacting their QoL compared to men. Data showed that white respondents presented better QoL scores (total and domains) than brown and black respondents. On average, black and brown Brazilians earn half the income of white individuals [27] and have limited access to healthcare services, negatively affecting their QoL. Data also suggests that individuals aged ≥40 y/o presented better QoL for all domains. Individuals with partners presented best QoL scores (except for the social domain—which was similar) than the ones without a partner, probably because marriage is associated with higher life satisfaction and happiness, related to better mental and physical health, emotional well-being and QoL and longevity [28,29,30]. Having children did not influence respondents general QoL. However, individuals with children presented a lower QoL perception regarding the economic domain than those without children. These results are most likely due to a larger household (children), generating additional expenses, such as water, energy, food, and other bills during the quarantine. 

A higher educational level is frequently associated with a higher perception of QoL. A study by Ryff and Heidrich, 1997 [31] showed that work and education experiences change life’s purpose. Higher education levels are associated with happiness and satisfaction and strongly affect income [32], potentially explaining our results. Unemployed individuals presented the lowest QoL scores (total and in each domain) explained by the economic burden that affects all QoL aspects, mainly during isolation, and the increase in household bills. Uncertainties about the future during a challenging economic period, such as future work conditions and employment, income, and social safety net, tend to lower the mean scores for QoL [8].

Our data showed that the four domains presented a good fit in the confirmatory factor analysis (RMSEA = 0.0068 (95% CI: 0–0.0157); CFI = 0.9992 and chi2 = 109.63; df = 101; *p* = 0.262), which means that the measures of the constructs are consistent with the understanding of their nature [14,16]. The total score Cronbach alpha values for psychological, social, and economic domains scores were >0.6, demonstrating good internal consistency. However, this study presents some limitations. First, despite a large number of participants, a convenience sample was used, which makes it harder to generalize the results. On the other hand, utilizing an online survey during a pandemic made it possible to reach individuals in every region of the country. Data from the latest survey published by the Brazilian Institute of Geography and Statistics showed that three out of four Brazilians in metropolitan areas (most of our participants) have internet access [33]. Therefore, although web-based research might be limited because it is impossible to reach a portion of the population, it can still be considered a viable strategy, since our questionnaire could be answered on any device. Moreover, our sample was composed mainly of females (72%), which can also be seen as a limitation. However, females tend to be more concerned about their health and participate in health surveys more frequently than males [34,35].

Another potential limitation is that it is a cross-sectional study and lacks pre-COVID-19 data for comparison; however, the data can be used for future comparisons. Although, social and family support is considered necessary in QoL study during the pandemic [8], it was not measured in this study.

## 5. Conclusions

Pandemics have historically posed a challenge for governments and individuals. During the pandemic, Brazilian’s QoL was more affected by economic and social aspects than psychological and physical. Having children (or not) and place of residence (urban or rural areas) did not influence the total QoL score. The psychological and physical domains were not affected by the educational level, place of residency, or whether or not respondents had children or not. The social domain was not affected by having children (or not), and place of residency, a family member diagnosed with COVID-19, or by marital status. The economic domain was not influenced by gender, place of residency, or by individuals who test positive or not for COVID-19. Further studies should be conducted to evaluate how the length of isolation affected participants and compare the period during and after the COVID-19 pandemic period.

## Figures and Tables

**Table 1:** Quality of life by socioeconomic and demographic variables of Brazilian individuals during the pandemic period.

Sub-Scores and Scale Subcategorized by the Characteristics of Participants	Psychological		Social		Physical		Economic		Total	
	Mean (SD)	*p*	Mean (SD)	*p*	Mean (SD)	*p*	Mean (SD)	*p*	Mean (SD)	*p*
Gender *										
Female (*n* = 1349)	15.20 (3.88) ^a^	0.000	14.63 (3.42) ^a^	0.001	17.44 (2.99) ^a^	0.000	14.51 (4.92) ^a^	0.060	61.79 (11.68) ^a^	0.000
Male (*n* = 510)	16.42 (3.49) ^b^		15.22 (3.31) ^b^		18.08 (2.84) ^b^		14.99 (5.06) ^a^		64.71 (10.88) ^b^	
Age *										
<40 y/o (*n* = 933)	14.74 (3.77) ^a^	0.000	14.52 (3.30) ^a^	0.000	17.07 (2.98) ^a^	0.000	14.02 (4.96) ^a^	0.000	60.34 (11.32) ^a^	0.000
≥40 y/o (*n* = 921)	16.34 (3.69) ^b^		15.08 (3.47) ^b^		18.17 (2.85) ^b^		15.27 (4.88) ^b^		64.85 (11.33) ^b^	
Marital status *										
Without partner (*n* = 933)	15.10 (3.89) ^a^	0.000	14.67 (3.42) ^a^	0.092	17.37 (2.96) ^a^	0.000	14.03 (4.95) ^a^	0.000	61.17 (11.51) ^a^	0.000
With partner (*n* = 924)	15.97 (3.69) ^b^		14.93 (3.36) ^a^		17.87 (2.95) ^b^		15.25 (4.91) ^b^		64.02 (11.41) ^b^	
Ethnicity **										
White (*n* = 1096)	15.74 (3.82) ^a^		15.02 (3.40) ^a^		17.85 (3.02) ^a^		15.15 (5.03) ^a^		63.75 (11.72) ^a^	
Brown (*n* = 515)	15.20 (3.78) ^b^	0.005	14.41 (3.34) ^b^	0.001	17.37 (2.86) ^b;c^	0.000	13.96 (4.66) ^b^	0.000	60.93 (10.96) ^b;c^	0.000
Black (*n* = 144)	14.81 (3.67) ^b^		14.16 (3.15) ^b^		16.67 (2.73) ^b^		13.15 (4.94) ^b^		58.78 (10.63) ^b^	
Yellow/Indigenous (*n* = 45)	15.87 (3.68) ^a;b^		14.87 (3.63) ^a;b^		18.24 (2.84) ^a;c^		15.27 (4.93) ^a;b^		64.24 (11.79) ^a;c^	
Children living at home *										
No (*n* = 966)	15.40 (3.75) ^a^	0.103	14.88 (3.42) ^a^	0.265	17.63 (2.97) ^a^	0.804	14.87 (4.96) ^a^	0.024	62.80 (11.50) ^a^	0.414
Yes (*n* = 855)	15.70 (3.89) ^a^		14.71 (3.37) ^a^		17.60 (2.98) ^a^		14.35 (4.95) ^b^		62.35 (11.63) ^a^	
Place of residency *										
Urban area (*n* = 1771)	15.51 (3.81) ^a^	0.177	14.77 (3.39) ^a^	0.085	17.61 (2.97) ^a^	0.546	14.67 (4.97) ^a^	0.201	62.56 (11.55) ^a^	0.569
Rural area (*n* = 81)	16.10 (3.90) ^a^		15.43 (3.42) ^a^		17.81 (3.00) ^a^		13.95 (4.81) ^a^		63.30 (11.32) ^a^	
Educational level **										
High school (*n* = 169)	15.47 (4.54) ^a^		13.95 (3.81) ^a^		17.38 (2.96) ^a^		11.81 (5.00) ^a^		58.62 (12.36) ^a^	
Higher Education (*n* = 693)	15.28 (3.97) ^a^	0.063	14.67 (3.37) ^b^	0.000	17.52 (3.07) ^a^	0.190	13.79 (4.90) ^b^	0.000	61.26 (11.73) ^b^	0.000
Postgraduate (*n* = 993)	15.72 (3.56) ^a^		15.02 (3.32) ^b^		17.73 (2.89) ^a^		15.71 (4.69) ^c^		64.19 (10.98) ^c^	
Professional occupation **										
Unemployed (*n* = 130)	14.25 (4.03) ^a^		14.02 (3.57) ^a^		16.68 (2.89) ^a^		10.19 (4.30) ^a^		55.14 (10.94) ^a^	
Student or intern (*n* = 256)	14.27 (3.79) ^a^	0.000	14.50 (3.28) ^a;b^	0.017	17.05 (2.99) ^a;b^	0.000	13.76 (4.75) ^b^	0.000	59.58 (11.07) ^b^	0.000
Government employee (*n* = 563)	15.73 (3.63) ^b^		14.89 (3.26) ^b^		17.66 (2.97) ^b;c^		16.41 (4.38) ^c^		64.70 (10.93) ^c^	
Others (*n* = 895)	15.94 (3.80) ^b^		14.91 (3.47) ^b^		17.87 (2.93) ^c^		14.43 (4.97) ^b^		63.15 (11.58) ^c^	
Positive COVID-19 *										
No (*n* = 1800)	15.58 (3.80) ^a^	0.000	14.83 (3.40) ^a^	0.003	17.66 (2.95) ^a^	0.000	14.66 (4.96) ^a^	0.215	62.73 (11.50) ^a^	0.001
Yes (*n* = 50)	13.54 (4.13) ^b^		13.36 (3.19) ^b^		16.00 (3.08) ^b^		13.78 (5.00) ^a^		56.68 (11.65) ^b^	
Family member positive COVID-19 *										
No (*n* = 1385)	15.76 (3.79) ^a^	0.002	14.89 (3.41) ^a^	0.367	17.74 (2.93) ^a^	0.037	14.70 (4.98) ^a^	0.788	63.09 (11.39) ^a^	0.048
Yes (*n* = 400)	15.10 (3.77) ^b^		14.71 (3.31) ^a^		17.37 (3.13) ^b^		14.63 (4.92) ^b^		61.81 (11.55) ^b^	
Social isolation **										
No social distancing (*n* = 33)	17.24 (3.65) ^a^		15.97 (3.42) ^a^		18.64 (3.66) ^a^		15.03 (5.31) ^a;b^		66.88 (12.63) ^a^	
Out only for essential purchase, working and visiting family (*n* = 237)	16.05 (3.81) ^a;b^		15.41 (3.52) ^a;b^		17.77 (3.06) ^a;b^		14.46 (4.95) ^a;b^		63.70 (11.92) ^a;b^	
Goes out only for essential purchase, but some family members go out to work (*n* = 698)	15.15 (3.78) ^b^	0.001	14.33 (3.28) ^b^	0.000	17.38 (2.92) ^b^	0.035	13.98 (4.96) ^a^	0.000	60.85 (11.02) ^b^	0.000
Goes out only for essential purchase (*n* = 760)	15.68 (3.75) ^b^		14.96 (3.43) ^a;b^		17.68 (2.92) ^a;b^		15.03 (4.88) ^a;b^		63.34 (11.55) ^a;b^	
Everyone stays at home and purchases are made online (*n* = 125)	15.30 (4.23) ^b^		14.82 (3.33) ^a;b^		17.90 (3.03) ^a;b^		16.10 (4.94) ^b^		64.13 (12.14) ^a;b^	

Note: Some variables have a sum less than *n* = 1859, as some individuals did not inform their data; * T-Student test; ** ANOVA with Tukey post-hoc test; Different letters (a, b, c) on the same column represent statistical differences (*p* < 0.05).

**Table 2:** The precision of the questionnaire and its domains (*n* = 1859).

Domain	Mean (SD) *	Median (IQR)	Range	Floor Effect (%)	Ceiling Effect (%)	Internal Consistency (Cronbach Alpha)
Psychological	15.54 (3.81) ^a^	16 (13–18)	5–25	0.8	0.3	0.728
Social	14.79 (3.40) ^b^	15 (12–17)	5–25	0.1	0.1	0.631
Physical	17.62 (2.97) ^c^	18 (16–20)	5–25	0	0.4	0.424
Economic	14.64 (4.96) ^b^	15 (11–18)	5–25	3.4	1.4	0.754
Total Score	62.59 (11.54)	63 (55–71)	27–96	0	0	0.837

* One-way repeated measures ANOVA followed by Bonferroni’s post-hoc tests. Different letters (a, b, c) on the same column represent statistical differences (*p* < 0.05).

## Supplementary Materials

The following are available online at https://www.mdpi.com/1660-4601/17/22/8554/s1, Table S1: Positive COVID-19 by QoL scores and socioeconomic and demographic variables of Brazilian individuals during the pandemic period.

## Appendix A. SurveyMonkey Questionnaire

Translated from Portuguese into English for publication.

This research aims to unveil how the pandemic caused by the new coronavirus and social isolation have affected the Brazilian quality of life. Your participation is very important!

All the data collected in this research will be used only by the research team and for this study. All the data will be studied protecting your identity, that is, anonymously and confidentially. Your name will not appear or be disclosed, and we ensure, once again, that the strictest confidentiality standards will be maintained given that the complete omission of any information that may identify you will not be available to anyone other than the research team.

The results of this research may bring important scientific information to assist in the development of public health actions for the benefit of the Brazilian population.

1. Do you agree to participate in this survey?
  Yes
  No
2. What is your biological sex?
  Feminine
  Male
3. How old are you (years)?
  (OVER 18 years old)
4. What ethnicity do you identify with?
  White
  Indigenous
  Black
  Brown
  Asian
  I would rather not answer this question
5. What is your marital status?
  Single
  Married/stable relationship
  Separated
  Divorced
  Widowed
6. How many children live and depend on you?
7. What state do you live in?
8. Your home is located in:
  Rural area
  Urban area
  Indigenous community
  Quilombola community
9. What is your level of education?
  No formal education
  From 1st to 4th grade of elementary school
  From 5th to 8th grade of elementary school
  High school
  Incomplete High School
  Incomplete higher education
  Complete higher education
  Post-Graduation-Specialization
  Post-Graduation- Master/Doctorate
  Post-Doctorate
10. What is your occupation?
  Student or intern;
  Employed in a private or mixed economy company
  Steady civil servant (federal, state, municipal)
  Small business owner
  Moderate/Big company owner
  Executive (?)
  Unemployed
11. Do you work in any of the professions listed below?
  Doctor
  Nurse
  Pharmaceutical
  Nutritionist
  Psychologist
  Nursing technician
  Laboratory technician
  Police Officer
  Firefighter
  Others
12. What is your average monthly income?
  No income
  Less than 1 minimum wage
  Up to 1 minimum wage
  From 1 to 2 minimum wages
  From 2 to 3 minimum wages
  From 3 to 5 minimum wages
  More than 3 to 5 minimum wages
  More than 5 to 10 minimum wages
  More than 10 to 20 minimum wages
  More than 20 minimum wages
13. Have you tested positive for COVID-19 (fast or PCR)?
  Yes
  No
14. Has anyone in your family been infected with COVID-19?
  Yes—They live with me
  Yes but they don’t live with me
  No
15. Have you and your family adhered to social distancing?
  Yes, everyone stays at home and purchases are made online
  Yes, we only go out for the essentials
  Yes, we only go out for the essentials, but some members leave the house to work
  Yes, we only go out for the essentials, work and visit some family members
  No, we have not adhered to social distancing measures
16. As a result of the coronavirus crisis, have you felt negative feelings such as anxiety, depression, despair? How often do you feel it?
  Always
  Very often
  Often
  Sometimes
  Never
17. How would you rate your quality of life during this period of social distancing (including sleep, sexual activity, etc.)?
  Very bad
  Bad
  Neither good nor bad
  Good
  Very good
18. How often, during the past two weeks, have you worried or been afraid of contracting COVID-19?
  Always
  Very often
  Often
  Sometimes
  Never
19. How concerned are you about the current situation and the possible outcomes of the pandemic caused by COVID-19?
  Extremely concerned
  Very concerned
  Moderately concerned
  Not very concerned
  Not concerned at all.
20. Have you been able to enjoy yourself during the social distancing period. How often?
  Never
  Sometimes
  Often
  Very often
  Always
21. I have opportunities for leisure activity during the social distancing.
  Strongly disagree
  Partially disagree
  I do not agree nor disagree
  Partially agree
  I fully agree
22. How satisfied are you with your personal relationships (friends, relatives, acquaintances, colleagues) during the period of isolation?
  Very unsatisfied
  Dissatisfied
  Neither satisfied/nor dissatisfied
  Satisfied
  Very satisfied
23. Are you concerned that one or more members of your family may be hospitalized? Do fear you losing contact with them due to isolation?
  Extremely concerned
  Very worried
  Moderately concerned
  Not too worried
  Nothing worried
24. How healthy is your physical environment (climate, noise, pollution, attractions) in this period of isolation?
  Not healthy at all
  Not very healthy
  More or less healthy
  Very Healthy
  Extremely Healthy
25. Are you concerned that, if necessary, you might not get adequate medical/hospital care for your treatment?
  Extremely concerned
  Very concerned
  Moderately concerned
  Not too concerned
  Not concerned at all
26. How satisfied are you with your health in this period of social distancing?
  Very unsatisfied
  Dissatisfied
  Neither satisfied/nor dissatisfied
  Satisfied
  Very satisfied
27. How often during the pandemic did you need to visit a health professional?
  Never
  Sometimes
  Often
  Very often
  Always
28. I have enough stamina and disposition for my regular routine during this period of social distancing.
  Not at all
  Very little
  Moderate
  Quite a lot
  A lot
29. My consumption of alcohol and or drugs increased during the social distancing period.
  Strongly disagree
  Partially disagree
  I do not agree nor disagree
  Partially agree
  I strongly agree
30. Neither me nor someone in my family, lost their job due to the crisis caused by the pandemic.
  Strongly disagree
  Partially disagree
  I do not agree nor disagree
  Partially agree
  I strongly agree
31. I have enough money to meet all my basic and family needs during this period of crisis.
  Strongly disagree
  Partially disagree
  I do not agree nor disagree
  Partially agree
  I strongly agree
32. I am concerned about my financial situation.
  Extremely concerned
  Very concerned
  Moderately concerned
  Not too concerned
  Not concerned at all
33. I have everything I need and I am not worried about my finances or my family’s future.
  Strongly disagree
  Partially disagree
  I do not agree nor disagree
  Partially agree
  I strongly agree
34. Despite the pandemic, I could handle a large unexpected expense.
  Strongly disagree
  Partially disagree
  I do not agree nor disagree
  Partially agree
  I strongly agree
