# Supplementary material for: Repercussion of COVID-19 Pandemic on Brazilians’ Quality of Life: A Nationwide Cross-Sectional Study
Source: Int J Environ Res Public Health. 2020 Nov 18;17(22):8554. doi: 10.3390/ijerph17228554 (PMC7698925; doi:10.3390/ijerph17228554)
Supplement: Supplementary file 1 [file ijerph-17-08554-s001.pdf]

Supplementary Material

Table S1. Positive COVID-19 by quality of life (QoL) scores and socioeconomic and demographic variables of Brazilian individuals during the pandemic period.

	Positive COVID-19		
	**No (n = 1800)**	**Yes (n = 50)**	p
**Gender (freq.; %)**			
Female [a]	1305 (97.2%)	38 (2.8%)	0.634 **
Male [a]	495 (97.6%)	12 (2.4%)	
**Age (freq.; %)**			
< 40 y/o [a]	902 (97.2%)	26 (2.8%)	0.886 **
≥ 40 y/o [a]	893 (97.4%)	24 (2.6%)	
**Marital status (freq.; %)**			
Without partner [a]	909 (97.6%)	22 (2.4%)	0.392 **
With partner [a]	889 (96.9%)	28 (3.1%)	
**Ethnicity (freq.; %)**			
White [a]	1065 (97.7%)	25 (2.3%)	
Brown [a]	492 (95.7%)	22 (4.3%)	0.122 ***
Black [a]	142 (98.6%)	2 (1.4%)	
Yellow/Indigenous [a]	44 (100%)	0 (0%)	
**Children living at home (freq.; %)**			
No [a]	943 (97.7%)	22 (2.3%)	0.185 **
Yes [a]	820 (96.7%)	28 (3.3%)	
**Place of residency (freq.; %)**			
Urban area [a]	1714 (97.3%)	48 (2.7%)	0.890 **
Rural area [a]	79 (97.5%)	2 (2.5%)	
**Educational level (freq.; %)**			
High school [a]	166 (98.2%)	3 (1.8%)	
Higher Education [a]	679 (98.7%)	9 (1.3%)	0.005 **
Postgraduate [b]	951 (96.2%)	38 (3.8%)	
**Professional occupation (freq.; %)**			
Unemployed [a]	126 (97.7%)	3 (2.3%)	
Student or intern [a]	250 (97.7%)	6 (2.3%)	0.213 **
Government employee [a]	537 (96.1%)	22 (3.9%)	
Others [a]	872 (97.9%)	19 (2.1%)	
**Social isolation (freq.; %)**			
No social distancing [a]	33 (100%)	0 (0%)	
Out only for essential purchase, working and visiting family [a]	228 (96.2%)	9 (3.8%)	
Goes out only for essential purchase, but some family members go out to work [a]	675 (97.3%)	19 (2.7%)	0.715 ***
Goes out only for essential purchase [a]	736 (97.5%)	19 (2.5%)	
Everyone stays at home and purchases are made online [a]	122 (97.6%)	3 (2.4%)	
**QoL Scores (mean; SD)**			
Psychological	15.58 (3.80) [A]	13.54 (4.13) [B]	0.000
Social	14.83 (3.40) [A]	13.36 (3.19) [B]	0.003
Physical	17.66 (2.95) [A]	16.00 (3.08) [B]	0.000
Economic	14.66 (4.96) [A]	13.78 (5.00) [B]	0.215
TOTAL	62.73 (11.50) [A]	56.68 (11.65) [B]	0.001

Note: Some variables have a sum less than $n = 1859$, as some individuals did not inform their data; * T-Student test; ** Pearson chi-squared test; *** Monte Carlo based chi-squared test; Different small letters (a, b, c) on the same column represent statistical differences ($p < 0.05$); Different capital letters (A, B) on the same line represent statistical differences ($p < 0.05$).
